# Alien Invasions and the Game of Hide and Seek in Patagonia

**DOI:** 10.1371/journal.pone.0044350

**Published:** 2012-10-10

**Authors:** Martin Lindegren, Pablo Vigliano, P. Anders Nilsson

**Affiliations:** 1 Scripps Institution of Oceanography, University of California, La Jolla, California, United States of America; 2 Univ Nacl Comahue, Ctr Reg Univ Bariloche, Bariloche, Rio Negro, Argentina; 3 Department of Biology – Aquatic ecology, Lund University, Lund, Sweden; University of Utah, United States of America

## Abstract

The introduction, establishment and spread of alien species is a major threat to biodiversity and the provision of ecosystem services for human wellbeing. In order to reduce further loss of biodiversity and maintain productive and sustainable ecosystems, understanding the ecological mechanisms underlying species invasions and avoiding potentially harmful effects on native communities is urgently needed, but largely lacking. We here demonstrate, by means of hydroacoustics and advanced spatial modelling, how native fish species as a result of previous exposure to native predators may successfully respond to invasive novel predators through a complicated game of hide and seek, minimizing spatio-temporal overlap with predators, and potentially facilitating coexistence between native prey species (Galaxiids) and introduced novel predators (Salmonids) in a deep Andean lake, Patagonia.

## Introduction

The introduction of alien species, deliberate or unintentional, is one of the main threats to biodiversity and the provision of ecosystem goods and services worldwide [1], [2]. Combined with other natural and anthropogenic disturbances, e.g., habitat destruction, overexploitation and climate change, invasive species take part in a process of global change affecting multiple ecological processes from individuals to the structure and functioning of ecosystems [3], [4]. Due to an increasing demand for fish products, mainly from aquaculture and recreational fishing, native fish communities are severely impacted by predation, competition, hybridization and disease transmission from introduced species [5]. To reduce further loss of biodiversity and maintain productive and sustainable aquatic ecosystems, understanding the ecological mechanisms of species invasions is therefore of vital importance [5], [6].

**Figure 1:**
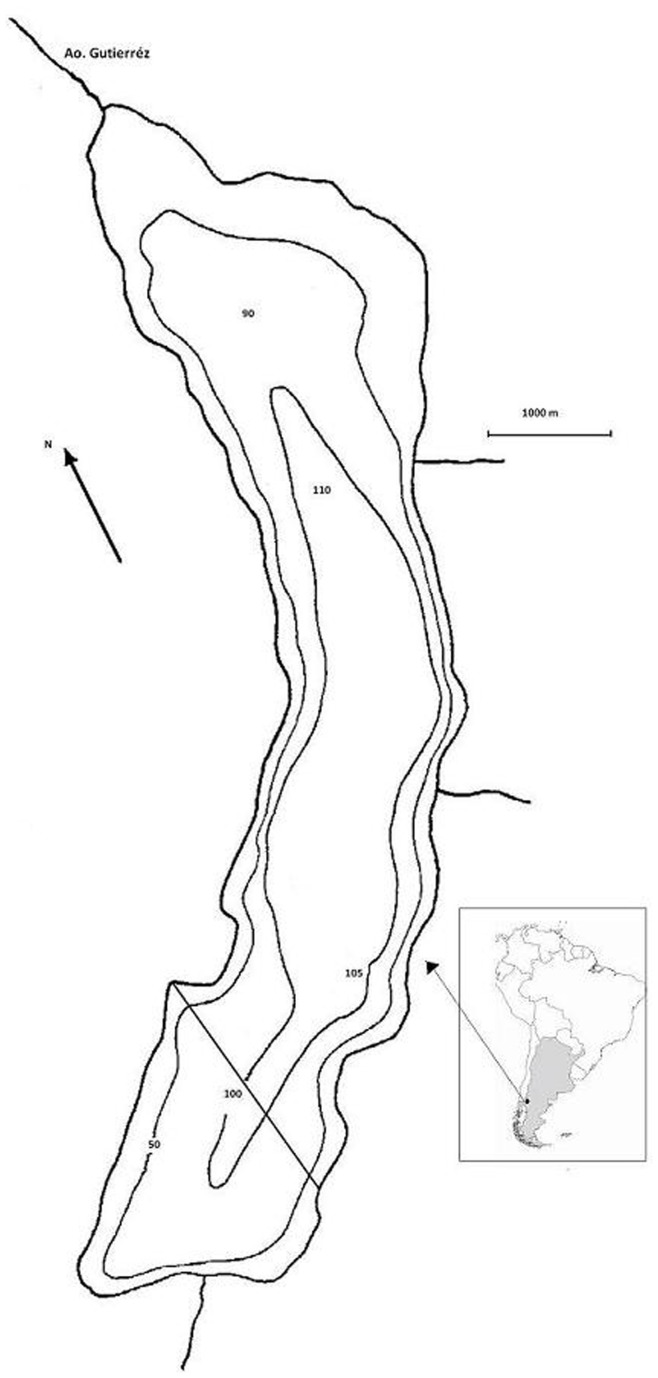
Map showing Lake Gutiérrez, depth contours (m) and the position of the transect between the western and eastern shore in the southern part of the lake.

Fish communities in Patagonia are generally low in species richness, due to little time for colonization or *in situ* speciation to have occurred in these young, post-glacial ecosystems [7]. During the past century, introduced salmonids have successfully established and spread, shaping native communities through predation and competition with local species [8]. Lake Gutiérrez is a deep ultra-oligotrophic lake [9], situated in the Southern Andes, Argentina (Figure 1). Due to high water clarity and scarcity of littoral refuge habitats (vegetation), the introduced predators, brown trout (*Salmo trutta*), rainbow trout (*Oncorhynchus mykiss*) and brook trout (*Salvelinus. fontinalis*), prey heavily on the native fish fauna [10]. Despite strong predation pressure, the main native prey, landlocked *Galaxias maculatus* (Jenyns) seems rather unaffected by salmonid introductions [10], occurring in high abundances with a widespread distribution range throughout the Southern hemisphere [11]. In contrast, many native galaxiid species in Australasia have responded drastically to salmonid introductions, causing extinctions, distribution shifts and cascading effects on lower trophic levels in many streams and lakes [12], [13]. Hence, a key issue is to identify what aspects of prey or predator biology explain why native prey sometimes exhibit ineffective antipredator behaviour and thus suffer heavy predation, whereas in other cases prey may detect and respond effectively to introduced predators [6].

We used high-resolution hydroacoustics, field sampling and statistical spatial distribution modelling [14] to investigate the daily distribution and habitat selection of prey and predatory fish in Lake Gutiérrez. Linked to recent theory on biotic invasions and predator-prey naïveté [6], [15], we here present evidence for a behavioural game of hide and seek [16] potentially enabling coexistence between native prey species and introduced predators.

## Materials and Methods

### Study Site Characteristics

Lake Gutiérrez lies within the National park of Nahuel Huapi in Northern Patagonia, Argentina (Figure 1). This deep and relatively large lake (i.e., mean depth of 80 m and an area of 16.4 km^2^) is monomictic and ultra-oligotrophic [17], [18]. Due to the high transparency (Secchi depth ∼20 m), light attenuates over a broad depth spectrum, causing an extended euphotic zone at depths between 40 to 50 m [19]. Hence, maximum primary production during summer occurs at or below the thermocline at depths around 40 m and constitutes almost entirely of mixotrophic ciliates [19], [20]. The zooplankton community is represented by the cladocerans *Bosmina longirostris, Ceriodaphnia dubia* and the calanoid copepod *Boeckella gracilipes* [9]. These species have shown to undertake pronounced diel vertical migration (DVM), mainly in response to hazardous UV-radiation [21], during night aggregating in high densities at depths corresponding to maximum primary production within the extended euphotic zone [21].

**Table 1:** Biotic and abiotic variables used as predictors during GAM model fitting.

Variable	Description	Abbreviation
Zooplankton availability	ln(Volume back scattering strength (dB))	Zpl
Predator ratio	N. of targets (TS = −20 to −40 db)/all targets	Pred
Depth	10 m depth intervals from 0–100 m	Depth
Distance	Distance to shore (m)	Dist
Hour	Time of day	Hour
Temperature	Vertical profile of temperature (C°)	Temp

**Figure 2:**
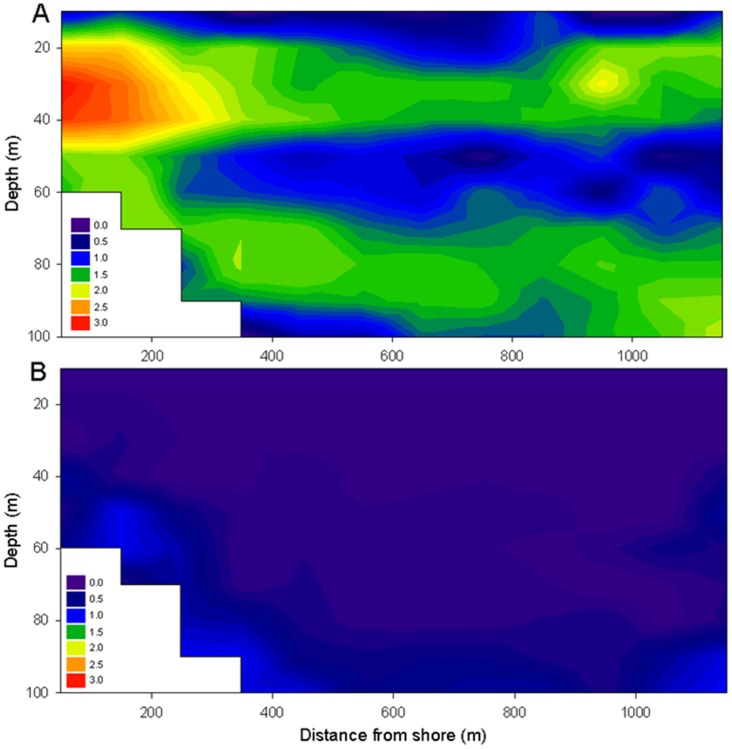
Daily fish distribution in Lake Gutiérrez. Average densities are high at night (A) and low during daytime (B). Peak densities (i.e., ln(N+1) ) are found near shore (20–40 m) and in lower densities along the bottom, consistent with habitat preferences of the main zooplanktivore *Galaxias maculatus*, and the less abundant, benthic relative *Galaxias platei* [25].

There is practically no information related to the composition of native fish communities before the introduction of salmonids (i.e., in the early 20^th^ century) or throughout the history of salmonid establishment. Although it is impossible to reconstruct the ichthyofauna before salmonid introductions, it is possible to assess the probable impact of salmonids on the predatory guilds in Patagonian lakes and reservoirs, based on the observed overlap in diet preferences and geographical distribution [22]–[24]. The fish community of Lake Gutiérrez is composed of the introduced salmonids, *Oncorhynchus mykiss*, *Salmo trutta* and *Salvelinus. fontinalis* and three native prey species, *Galaxias platei* (Steindachner), *Galaxias maculatus* and *Olivaichthys viedmensis* [25], [26].

**Table 2:** Fitting and validation summary of the final GAMs.

A. Fitting									B. Validation						
Nr.	Predictors	F/Chi.sq	DEV	r	a	b	r^2^ (%)	n	r	a	b	r^2^(%)	RMSE	AVE	n
I	Zpl, Pred, Depth, Dist, Hour	(261, 139, 194, 13, 11)	50.7	0.73	0.1	0.93	52.2	4359	0.7	0.3	0.99	49.3	0.66	−0.3	2286
II	Zpl, Pred, Depth, Dist, Hour	(554, 299, 482, 35, 17)	48.3	0.71	0.23	0.83	50.5	4359	0.69	0.32	0.93	48.2	0.66	−0.29	2286
III	Zpl, Pred, Depth, Dist, Hour	(214, 49, 30, 42, 8)	51.8	0.72	0	1	51.8	2184	0.76	0.4	0.89	57.1	0.5	−0.27	1238
IV	Zpl, Pred, Depth, Hour	(273, 248, 497, 34)	43.3	0.71	0	1	50	4359	0.68	0.19	0.97	46.2	0.41	−0.18	2286

The selected set of predictors, corresponding F/Chi-sq. values and total deviance explained (DEV) for each model, where models I-III use fish abundance estimates as response variables while model IV use presence-absence data only. Model fit and predictive accuracy was assessed by correlations (r) and linear regression between observed and fitted values (A), as well as between observed and predicted values (B), when fitted on only half the data set and predicting the remaining part. Finally, the explained variance (r^2^) of the linear regressions, the root mean square error (RMSE) and average error (AVE) between predicted and observed values, as well as the number of observations (n) are shown.

**Figure 3:**
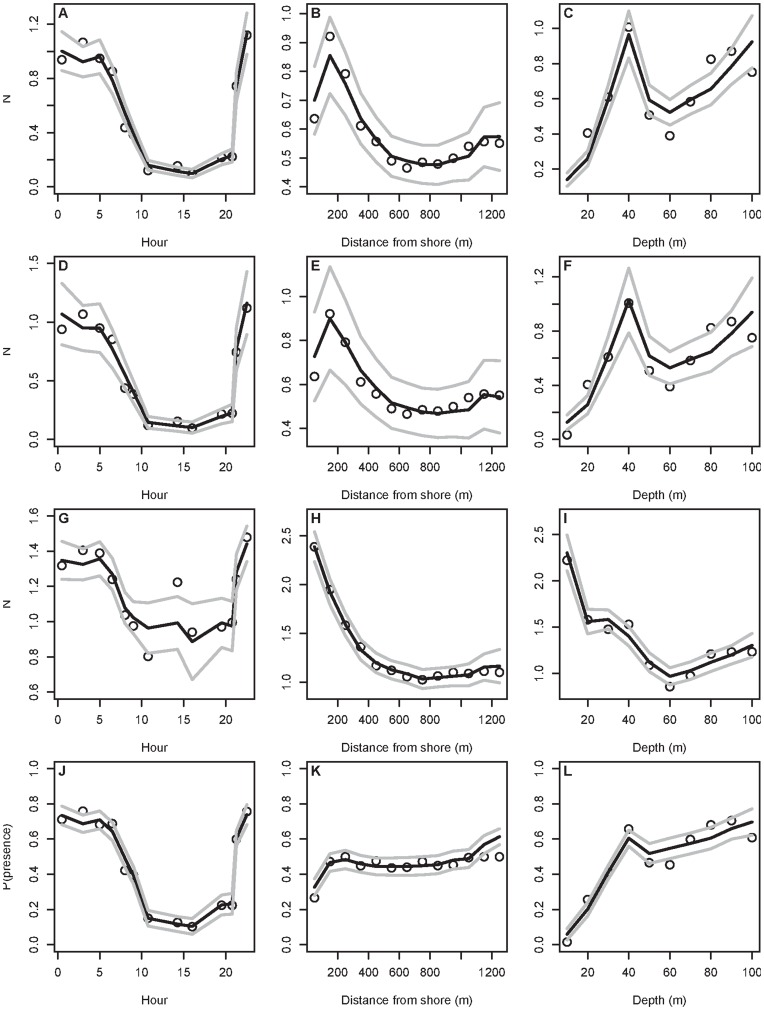
Predicted (black) and observed (circles) distribution of fish. Predictions are based on the final set of GAMs fitted to only a subset of the available data (day 1) and thereafter simulating predicted values to the remainder of the data set (day 2) by hour (left column), distance from shore (middle column) and depth (right column). Predictions originate from the final (A–C) Quasi-poisson GAM, (D–F) Negative-binomial GAM, (G–I) Gaussian GAM on presence data only, and (J–L) the Binomial GAM on presence-absence data, respectively. Upper and lower 95% prediction intervals are indicated by gray lines.

The present lack of native predators in Lake Gutiérrez, *Percichthys trucha* and *Odontesthes hatcheri* is likely due to diet overlap and competitive exclusion following salmonid introduction [10]. Nevertheless, Lake Gutiérrez exhibits a high incidence of piscivory, due primarily to high visibility and the virtual lack of vegetation and littoral refuge areas [10], [27]. Hence, the introduced salmonids feed extensively on *Galaxias maculatus*, but also a pronounced predation within the salmonids exists, mainly conducted by *Salmo trutta*. The native species feed predominately on the highly diverse benthos, and in the case of *Galaxias maculatus* largely on zooplankton [9], [28], [29].

**Figure 4:**
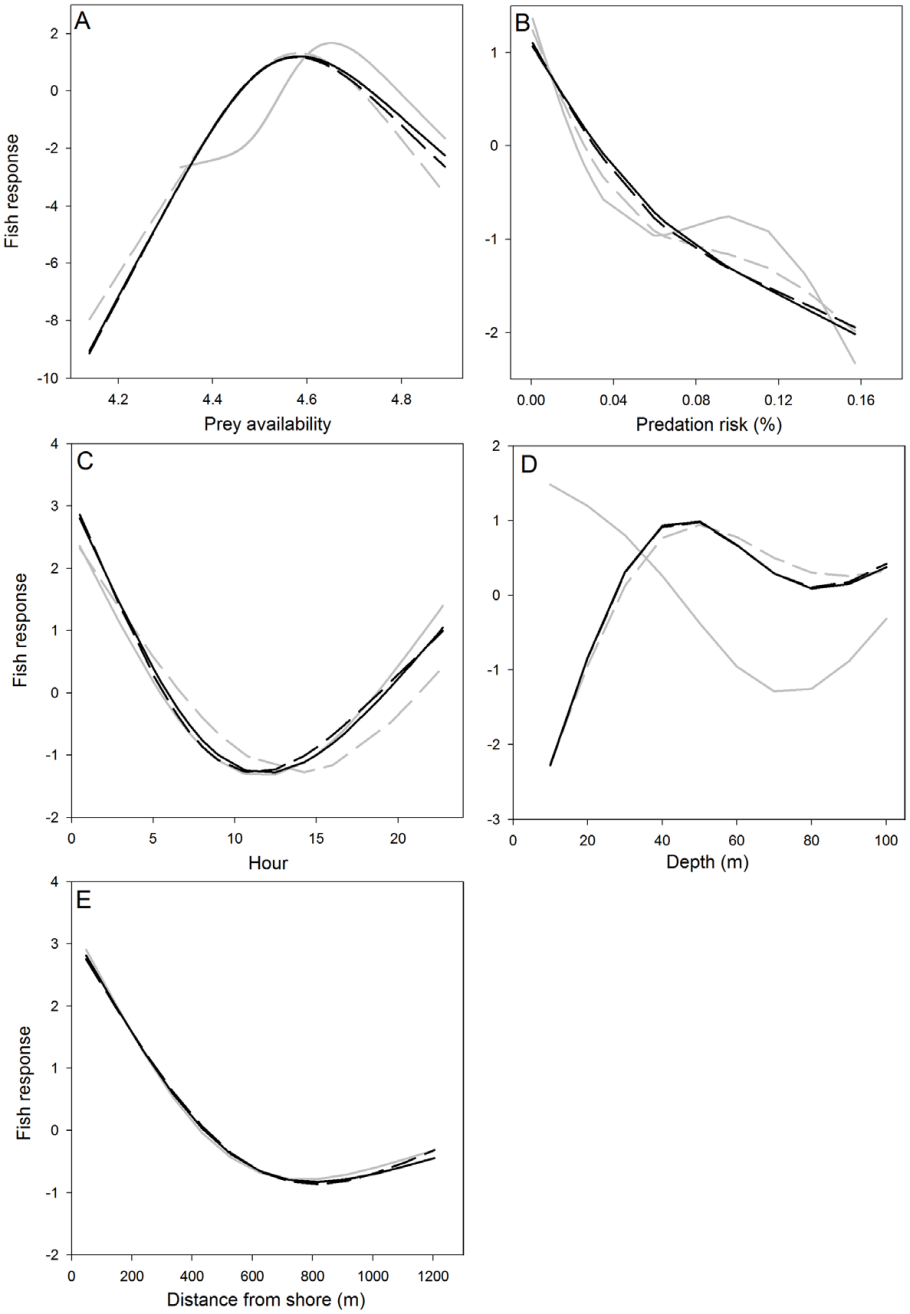
Functional relationships between fish density (fish response) and the final set of predictors. (A) prey availability, (B) predation risk, (C) time of day (hour), (D) depth and (E) distance from shore. The different smooth curves in each panel correspond to relationships from each model in the final set of GAMs, i.e., model I (black solid), II (black dashed), III (grey solid) and IV (grey dashed).

### Data Collection and Hydroacoustics

The investigation of fish distribution and migration in Lake Gutiérrez was conducted along a transect between the western and eastern shore during two separate 24-hour periods in March 2003. Two transducers, one single beam 208 kHz and one split beam 120 kHz transducer, were used simultaneously. This gives the advantage of simultaneously acquiring data on fish- and zooplankton distribution, as well as direct measurements of fish size, through target strength (TS) measurements [30]. Calibration was carried out *in situ* against the standardized acoustic properties of a 36 mm metal sphere deployed below the transducers. In addition to hydroacoustic data, physical zooplankton samples were obtained by two ichthyoplankton nets of mesh sizes 1500 µm (50.5 cm diameter, 260 cm length) and 270 µm (32 cm diameter, 105 cm length) towed vertically from the bottom during several occasions each night. The samples were instantly preserved in 4% formaldehyde solution.

**Figure 5:**
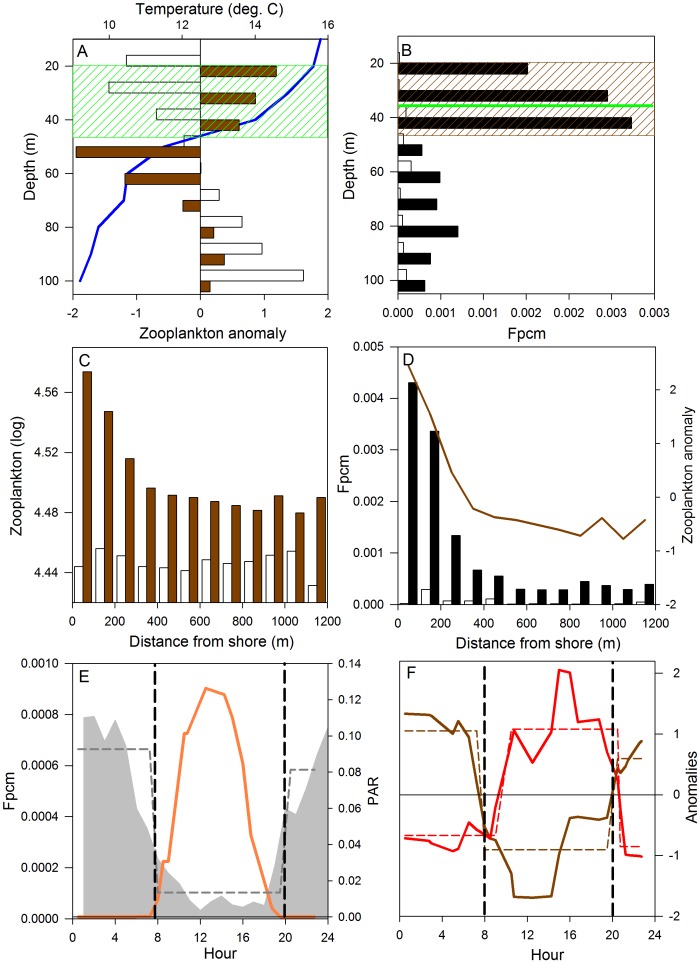
Summary of abiotic and biotic factors regulating the predator-prey game of hide and seek. (A) Zooplankton vertical distribution (night = brown, day = white), temperature (blue) and the extended euphotic zone (green). (B) The deep chlorophyll maximum [19] (green), zooplankton availability (brown) and average fish densities (fish per m^−3^) during night (black) and day (white). (C, D) Zooplankton and fish densities by distance from shore (day and night). (E) Average fish density (grey) and surface irradiance (PAR; orange) by hour. (F) Average prey availability vs. predation risk (anomalies), illustrating the trade-off between foraging and mortality, where the timing of sunrise and sunset (dashed vertical lines) coincide with significant thresholds (Supporting Information) in food availability (brown dashed), predation risk (red dashed) and fish density (E; grey dashed).

By combining physical sampling, including also prior gill-net surveys in the area [25], [26], with hydroacoustic data enables a validation of the derived distribution patterns of fish and zooplankton, and more importantly a sound ecological basis for interpretation of the underlying behavioral mechanisms. Finally, temperature measurements at different depths were collected, as well as daily measurements of surface irradiance (µE/cm^2^·sec) in the photosynthetically active radiation spectra (PAR), obtained from sampling records taken in the neighboring Lake Moreno of similar physically and ecologically properties [9], [17], [18]. All permits necessary for the described field studies were obtained from the National Park Administration of Argentina, through their technical office in San Carlos de Bariloche, Argentina.

### Data Handling and Processing

In order to filter out surface and bottom noise, the first meter below the transducers, the so-called transducer near zone, and the last 2.5 m above the bottom, the so-called bottom blanking zone, were excluded from further analysis. The larger bottom blanking zone was chosen due to disturbances during a few transects with windy conditions. To guarantee a standardised data processing the same settings were used for all transects. To compare distribution and daily migration patterns at ecologically relevant spatial scales, the acoustic backscattering (−dB), corresponding to the amount of reflected energy received at the transducer surface, was integrated vertically into depth strata of 10 m and horizontal sections of 100 m. The degree of spatial resolution was chosen based on prior knowledge on the vertical and horizontal distribution of fish from gill-net surveys [25], [26] and aimed to constrain data handling, analysis and computation time within reasonable limits.

The acoustic signal received by the split-beam transducer was filtered through a threshold interval of –20 to −58 dB, thereby excluding strong echoes from high density abiotic objects and weaker signals originating from smaller planktonic organisms including icthyoplankton (i.e., galaxiid larvae), thus yielding estimates of fish density (abundance) per bin. As an index of predation risk [31], we used the proportion of targets above −40 dB (Figure S3), corresponding to large, mainly piscivorous salmonids (i.e., lengths above ∼20 cm; [10]), different from targets below −40 dB, representative of the smaller native prey species [25]. Since juvenile salmonids remain primarily in the tributaries where they are spawned, the predation risk index mainly reflects the predation risk experienced by the native prey species in Lake Gutiérrez.

Single beam data on zooplankton distribution was filtered by the threshold interval of –90 to −70 dB (corresponding to sizes between 0.5 to 5 mm), thus excluding stronger echoes attributed to fish and early life-stages of fish, e.g., such as the planktonic larvae of *Galaxias maculatus* and *Galaxias platei* [25], [32], [33]. We used the mean volume backscattering strength directly, as an index of zooplankton availability [34]. This gives no exact estimation of zooplankton densities, but gives the possibility to compare distribution patterns over space and time, as well as between trophic levels. Therefore, the physical samples aid not only in the interpretation of the hydroacoustic data, by providing insight into the species composition of the zooplankton community, but give a rough measurement of average densities.

### Statistical Analysis and Spatial Modeling Using a Multi-model Approach

Despite recent developments in species distribution modelling, efforts are needed to strengthen the link between model practice and ecological theory, accounting for biotic interactions, improving model selection and evaluating model uncertainties [14]. To address these issues, we used a multi-model approach, based on modern nonlinear regression techniques, i.e., Generalized additive models (GAMs) allowing for flexibility for fitting ecologically realistic relationships [35], [36], to study the functional relationship between fish distribution and multiple abiotic and biotic variables. We applied an array of GAMs on counts, presence-absence data and presence data, derived from hydroacustic sampling of fish distribution. Due to an excess number of zero observations in the data set, we used (I) Quasi-poisson and (II) Negative binomial GAMs, capable of modelling zero-inflated and overdispersed data [37], [38]. In addition, we modelled presence-absence data using a (III) Binomial GAM, and the presence part separately by a (IV) Gaussian GAM [39]. The following model formulation was used for all models (I–IV):
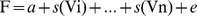
Where F is the response (i.e., fish abundance or presence/absence), *a* is the intercept, *s* the thin plate smoothing function [40], [41], V_i_….V_n_ a number of *a priori* selected biotic and abiotic predictors known to affect the spatio-temporal distribution of fish (Table 1) and ε the Gaussian error term. We applied a stepwise forward selection routine based on minimizing the generalized cross validation (GCV) and UBRE scores, and likelihood ratio tests to select the most parsimonious set of predictors for each model. The spline smoother function (*s*) was constrained to three degrees of freedom (k = 3), in order to allow for potential nonlinearities, but restrict large and unrealistic shapes of the resulting response functions for each biotic and abiotic predictor. Residuals were checked for normality, temporal- and spatial autocorrelation by means of Moran’s I and Mantel test [42] for each final model.

A way of validating the predictive capabilities of a model is to fit on a subset of the available data and then check the model by forecasting the remainder of the data. To that end, we fitted the final GAMs only to the first day of the data and used the derived functional relationships to simulate the second day. The simulated distribution patterns were then compared with the actual observed patterns of fish distribution for day two as to assess the predictive accuracy each model. All statistical analysis were conducted using the R software [43] version 2.12.1 (*mgcv* library) freely available at http://www.R-project.org).

## Results and Discussion

Despite large variability within and between sampled transects, hydroacoustic records demonstrate consistent patterns of fish distribution, with high abundances during night, low abundances during day and two periods of rapid increase and decrease at dusk and dawn, respectively (Figure 2, Figure S1, S2, Movie S1). This is consistent with recent findings from neighbouring lakes [33], suggesting that this distributional pattern is a widespread phenomenon throughout Northern Patagonia. Peak abundances in lake Gutiérrez are found near shore (>200 m) at 20–40 m depth, and in lesser numbers along the bottom (70–90 m). The density patterns and size distributions are consistent with previous gill-net, ichthyoplankton tows and baited trap studies on *Galaxias maculatus* [25], forming pelagic schools near shore at depths ∼30 m, and the deeper distribution of *Galaxias platei* [33], a larger relative morphologically and physiologically adapted to benthic life [11], [27]. The remaining native species, *Olivaichthys viedmensis*, a catfish species endemic to Patagonia [7], is found in low numbers close to the bottom [26], therefore contributing marginally to hydroacoustic records since it resides within the bottom blanking zone. Finally, single targets observed offshore during day (e.g., Figure S2A, Movie S1), showing little size overlap with native species [Figure S3], are likely caused by large rainbow, brown and brook trout, with decreasing depth preference in terms of vertical distribution, respectively [26].

After model fitting and selection (Table S1, Figure S4), the final GAMs demonstrate a high degree of explained variance and predictive accuracy in simulating the observed distribution patterns (Table 2, Figure 3). Since model residuals demonstrate no temporal or spatial autocorrelation (Table S2, Figure S5, S6) and robust predictions across models (Figure 3), the remaining uncertainty likely originates from other factors, such as hyperaggregation (schooling) or highly individual behaviour in space and time (e.g., Figure S2, Movie S1).

The functional relationships between fish distribution and the selected predictors show similar nonlinear interactions across models (Figure 4). All models show dome shaped responses to prey availability, with fish selecting patches of high, but not maximum, zooplankton densities (Figure 4A). Physical samples revealed dominance of cladocerans *Ceriodaphnia dubia*, *Bosmina longirostris* and the copepod *Boeckella gracilipes*, key prey items for *Galaxias maculatus* during summer [9]. Average densities and zooplankton composition conformed to previous studies [9], [21], demonstrating pronounced spatial aggregation (Figure 5A), where fish feeding rates are likely limited by pursuit and handling time, rather than encounter rates within local prey patches. The functional relationships with predation risk, estimated as the proportion of large targets (>20 cm), corresponding to the size distribution of native prey vs. introduced predators (Figure S3), demonstrate an exponentially decaying function overall, with declining densities or probability of presence, as the predation risk increases (Figure 4B).

All models show strong U-shaped relationships with time of day (Fig. 4C), reflecting high predation risks during daylight [10] and low food availability as zooplankton descend and disperse into deeper waters (Figure 5A), primarily to avoid hazardous UV-radiation [21]. Similarly, the functional relationships with depth illustrate avoidance of illuminated surface waters (Fig. 4D), where in case only presence is modelled local densities are high (grey solid; <1% of all observations found above 10 m), indicating potential schooling behavior in response to predation risk (Figure 3I). Fish mainly concentrate at depths ∼40 m, corresponding closely with the thermocline, the zone of maximum primary production [20], and the preferred depth distribution of zooplankton during night (Figure 5A, B). Furthermore, densities are higher near shore compared to the offshore areas (Figure 4E). Although not investigated in detail, zooplankton seem to aggregate near shore during night (Figure 5C), potentially due to stream discharge at the sample site with local enhancement of primary production (i.e., food availability) [44].

In order to summarize the combined effects of multiple abiotic and biotic factors on the spatio-temporal distribution patterns of fish (Figure 4), we outline the daily habitat selection of zooplankton, native prey fishes, *Galaxias maculatus* and *Galaxias platei,* and introduced predators as an intricate game of hide and seek [16] (Figure 5). During daylight zooplankton are dispersed in deep waters, avoiding high surface irradiance and hazardous UV-radiation [21], while native prey species take refuge from visual predators mainly close to the shore and bottom, hence avoiding detection from hydroacoustics by residing mainly below the bottom blanking zone. Large (piscivorous) salmonids are highly mobile predators, which according to gill net studies and hydroacoustic records are more abundant near shore and may occur openly in the pelagic zone [26], [33] while facing little risk of predation and cannibalism [10]. Juvenile salmonids minimize the risk of cannibalism by remaining in the tributaries where they are spawned or close to the shore, feeding on benthos [25], [26]. As light intensity decreases during dusk it triggers a rapid ascent of zooplankton which is accompanied by a swift horizontal migration of native prey fish (Figure 5E–F) to the near shore regions, thereby exposing themselves to potential predation from introduced salmonids. Night brings peak abundances of fish and dense aggregations of zooplankton (Figure 5A, B) centered near shore at the thermocline and the zone of maximum primary production [20], [21], as well as in lesser abundances along the bottom. These bottom dweller are likely composed of *Galaxias platei* [25], [26], physiologically and morphologically adapted to benthic life [27]. As the light intensity increases again during dawn, zooplankton quickly descend and disperse into deeper waters and fish show a reversed migration pattern, leaving the near shore regions for the relative safety of the shores.

We have demonstrated the trade-off between predator avoidance and foraging gain (Figure 5F), as an intricate game of hide and seek [16], involving biotic interactions between multiple trophic levels and abiotic factors related to light, temperature and bathymetry in Lake Gutiérrez (Figure 5). In line with recent studies of fish distribution in nearby Andean lakes [24], [33], our findings indicate that this would be a common behavioural process for deep ultraoligotrophic lakes in Patagonia. Below we interpret and discuss our findings in light of current theory on biotic invasions, concerning why native prey communities sometimes exhibit ineffective antipredator behaviour and thus suffer heavy predation and potential extinction, whereas in other cases, prey may detect, identify and respond effectively to introduced novel predators [6], [15].

Firstly, vacant or underutilized niches, in combination with low species richness, may explain community vulnerability to biotic invasions [45]. The young aquatic ecosystems of Patagonia are low in species richness [7]; yet native fish fauna demonstrates marked differences in response to invasions, from relatively marginal effects to competitive exclusion, distribution shifts and local extinction [8]. Although there is practically no information related to the composition of native fish communities before the introduction of salmonids, it cannot be inferred that the top predator niche was previously vacant because in many deep lakes throughout Patagonia the top predator niche was occupied by the native perch *Percichthys trucha*. It is also worth noting that *Odontesthes hatcheri*, a silverside endemic to Patagonia, acted as an additional native predator of galaxiid larvae [10], [27]. Therefore antipredator behaviour like the ones described for Lake Gutiérrez and the nearby Lake Moreno [33] may have evolved in relation to these native predators prior to salmonid introductions.

Secondly, natural or anthropogenic disturbances may promote invasions, by sudden and radical disturbances in the physical environment [4]. Andean lakes are naturally variable, experiencing volcanic activity, recurrent fires within the catchments, strong seasonality in temperature and precipitation [9], as well as anthropogenic effects from land use and hydropower. Despite these factors, native and introduced fish species occupy and partly coexist over a wide range of habitats, from almost pristine National parks to man-made dams and reservoirs [8]. Therefore, it seems unlikely that natural and anthropogenic disturbance could serve as a generalized explanation for successful establishment and spread of introduced salmonids in Patagonia.

Thirdly, the ‘prey naïveté hypothesis’, regards how a naïve prey, lacking evolutionary history with a non-native predator, may fail to recognize or respond adequately to predation, given by the similarity between non-native and native predators in terms of (i) spatio-temporal overlap (encounters), (ii) detection and recognition (e.g., visual or chemical cues) and (iii) the type of antipredator response [6]. Despite the present lack of native predators in Lake Gutiérrez, *Galaxias maculatus* and *Galaxias platei* have as explained earlier historically experienced predation from native *Percichthys trucha* and *Odontesthes hatcheri* [10], [27]. Therefore, native prey species in Lake Gutiérrez and Patagonia at large may have evolved antipredatory behaviour already before the introduction of salmonids. In contrast, many native galaxiid species in Australasia, e.g., landlocked koaro (*Galaxias brevipinnis*), have historically experienced no or few native predators [11], [13] therefore responding drastically to salmonid introductions, causing extinctions, distribution shifts and cascading effects on lower trophic levels in many streams and lakes [12], [13]. Interestingly *Odontesthes hatcheri* has diminished throughout Patagonia, probably due to active predation by salmonids despite its previous exposure to the native perch *Percichthys trucha*. However, *Odontesthes hatcheri* thrives in shallow lakes of the Patagonian plateau where it was introduced as fodder for salmonids. In the latter case its success over salmonids seems probably related to its active zooplanktivory causing increasing phytoplankton biomass, resulting in increased turbidity, reduced visibility and limited efficiency of salmonid predation in these shallow lakes.

Whereas the vacant niche, the disturbance and/or, the ‘prey naïveté hypothesis’ likely explain the contrasting patterns of extinction and coexistence between species in certain ecosystems, none of them by themselves seem enough to explain the coexistence pattern observed in Lake Gutiérrez or the present spread of salmonids and species interaction patterns found in deep ultraoligotrophic lakes throughout Patagonia. There are indications that resource partitioning and fish community structure vary in Patagonia in relation to abundance of *Percichthys trucha* [46]. Also historical stocking practices may produce shifts in invasion success. In Patagonia, initial introduction of *Salvelinus fontinalis* and *Salmo salar* was followed by stocking of *Oncorhynchus mykiss and Salmo trutta*. This produced dramatic shifts in species composition in freshwater bodies of Northern Patagonia [23], where even the previously introduced *Salmo salar* receded from most of its distribution range [23], [46]. Furthermore, differences in dispersal ability may influence invasion success and the degree of coexistence between native and introduced species [47]. While spatially explicit predator-prey dynamic models generally infer stability and coexistence within a larger meta-community based on a long-term balance between local extinction and colonization of prey and predators [48], the general lack of coexistence between native fish species and introduced predators throughout Australasia may be due to the relatively limited dispersal abilities of largely non-migratory galaxiids compared to the introduced salmonids which may rapidly disperse and colonize new habitats [47]. The role of dispersal barriers is illustrated by the nearby Lake Guillemo where a dam and waterfall hindered fish migration and consequently lead to stunted populations of *Salvelinus fontinalis* and *Oncorhynchus mykiss* [49].

Another factor that should be considered is the resilience of the native species themselves. *Galaxias maculatus* has undoubtedly adapted to the salmonid introductions in many environments, and it cannot be said that their distributional range in Argentine Patagonia [50] or abundance seem to have diminished. Thus, it seems likely that resilience due to behavioural (i.e., diel migrations patterns), physiological and reproductive characteristics of this species has ensured its adaptation to the introduction of salmonids.

Our results illustrate how native Galaxiid species in Patagonia may successfully reduce encounters with invasive salmonid predators through pre-existent adaptive behavioural responses, potentially facilitating coexistence through a complex game of hide and seek [16].Although largely different from theoretical population dynamic models of predators-prey interactions [48], our results on daily distribution patterns and behavioural aspects of habitat selection may provide insight into further developments of game theoretic approaches regarding hide and seek dynamics and predator-prey interactions in general [16], [51]. Furthermore, understanding the ecological mechanisms between introduced and native species is of vital importance in preventing future loss of biodiversity [27], and bridge the conflicting demands of nature conservation, aquaculture and recreational fishing in the area and beyond [5], [8], [52].

## Supporting Information

Figure S1. (PNG)

Figure S2. (PNG)

Figure S3. (PNG)

Figure S4. (PNG)

Figure S5. (PNG)

Figure S6. (PNG)

Table S1. (DOC)

Table S2. (DOC)

Text S1. (DOC)

Movie S1. (MPG)
