# Supplementary figures and images for: Alien Invasions and the Game of Hide and Seek in Patagonia
Source: PLoS One. 2012 Oct 10;7(10):e44350. doi: 10.1371/journal.pone.0044350 (PMC3468591; doi:10.1371/journal.pone.0044350)

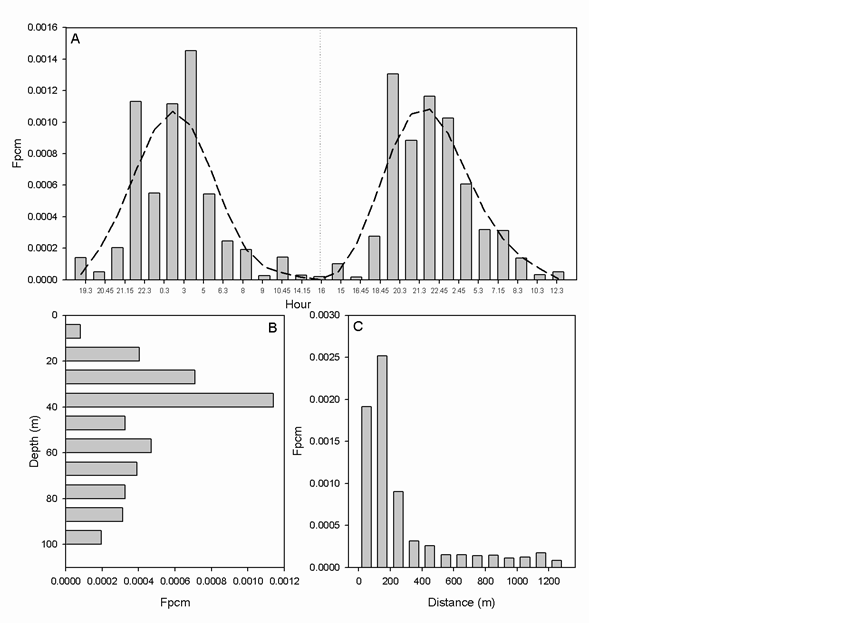

Supplement: Figure S1. — (PNG) [file pone.0044350.s001.png]

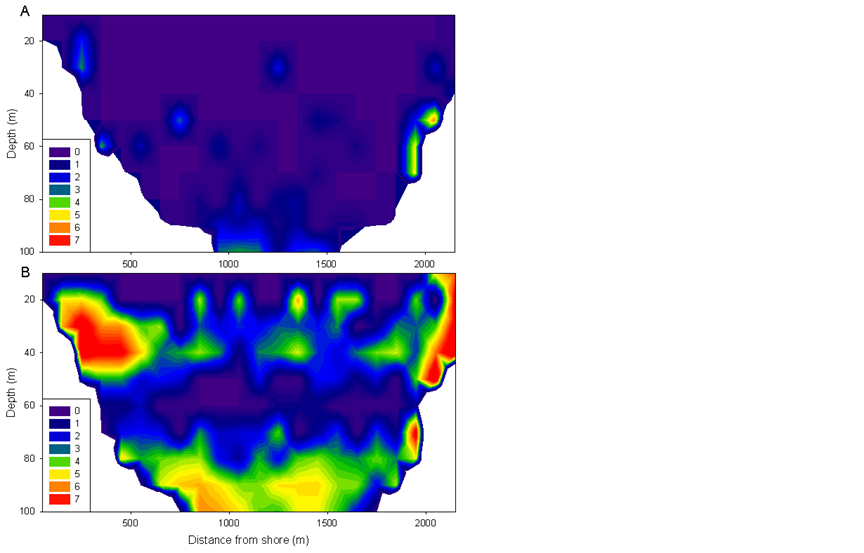

Supplement: Figure S2. — (PNG) [file pone.0044350.s002.png]

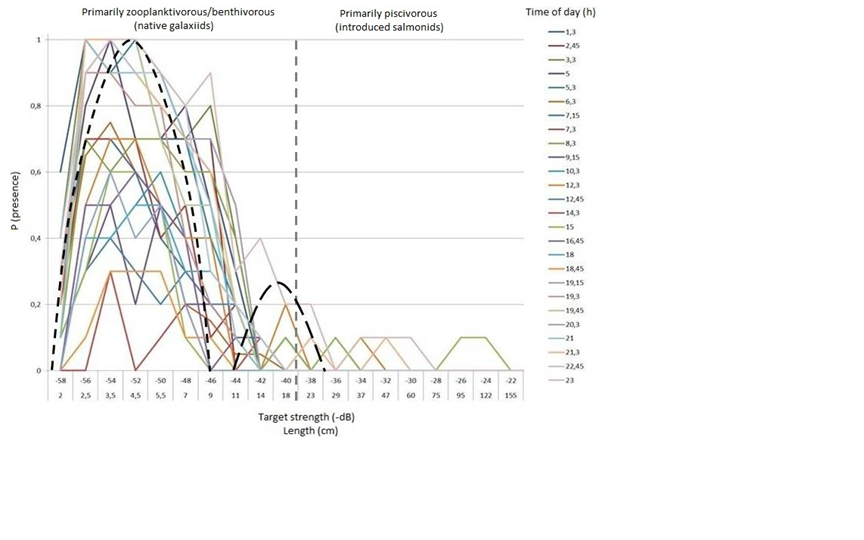

Supplement: Figure S3. — (PNG) [file pone.0044350.s003.png]

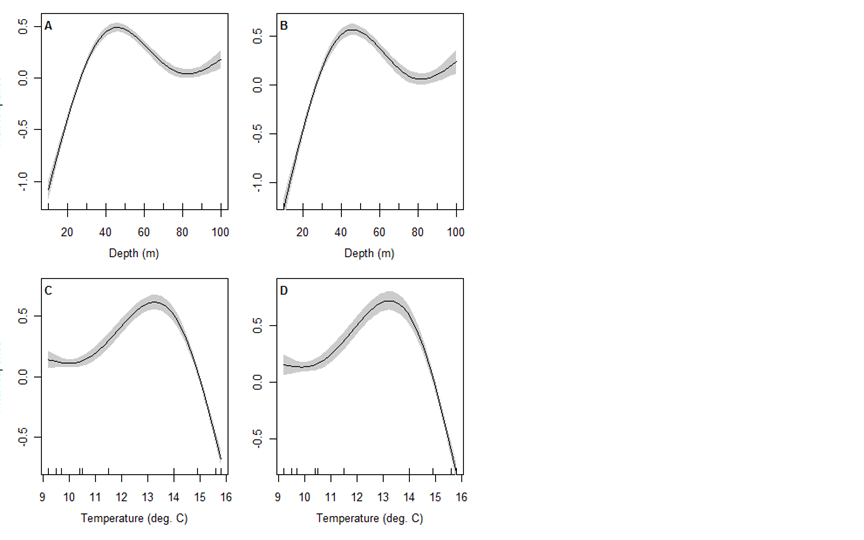

Supplement: Figure S4. — (PNG) [file pone.0044350.s004.png]

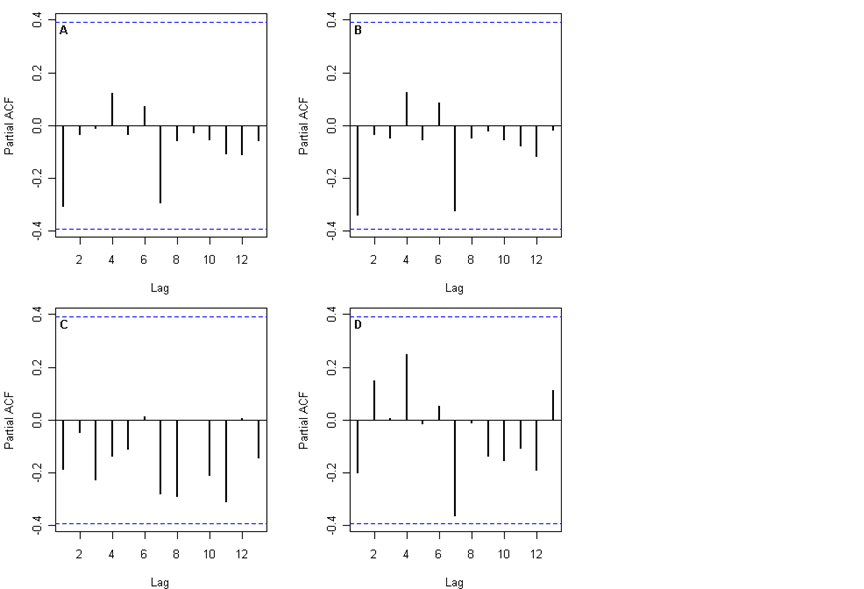

Supplement: Figure S5. — (PNG) [file pone.0044350.s005.png]

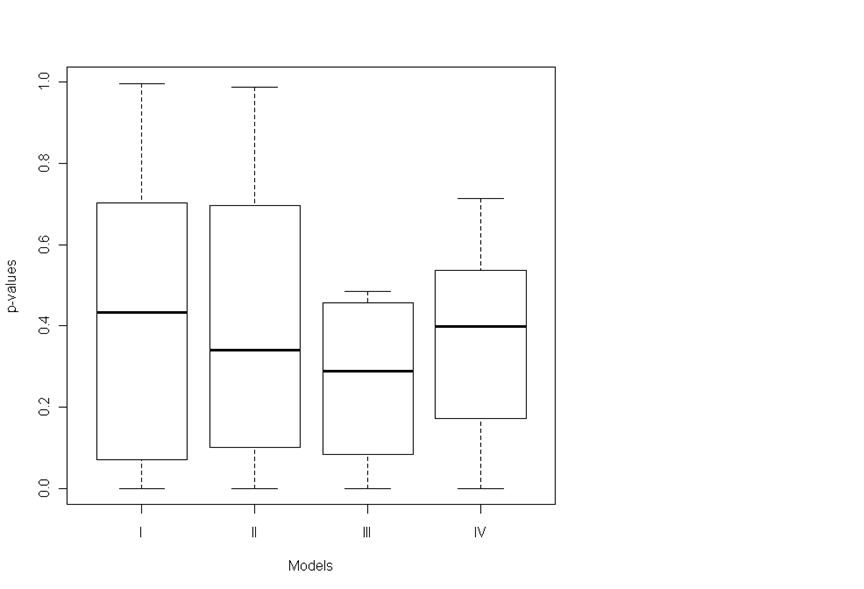

Supplement: Figure S6. — (PNG) [file pone.0044350.s006.png]
