# Supplementary material for: Alien Invasions and the Game of Hide and Seek in Patagonia
Source: PLoS One. 2012 Oct 10;7(10):e44350. doi: 10.1371/journal.pone.0044350 (PMC3468591; doi:10.1371/journal.pone.0044350)
Supplement: Table S1. — (DOC) [file pone.0044350.s007.doc]

**Table S1. Forward model selection based on likelihood ratio tests and GCV/UBRE scores for each GAM (I-IV).** The total deviance explained (DEV in %), the significance of the likelihood ratio test (P) and the number of observations (n) are shown. Final models are highlighted in bold. (Temperature was excluded due to colinearity with depth and identical functional relationship; Figure S4). (I=Quasi-poisson; II=Negative-binomial; III=Gaussian on presence data only, IV=Binomial on presence-absence data)

| I. Quasi-poisson GAM |  |  |  |  |
| --- | --- | --- | --- | --- |
| Nr Predictors | GCV/UBRE | DEV (%) | P | n |
| 1 Fish ~ Zpl | 1.738 | 30 |  |  |
| 2 Fish ~ Zpl + Pred | 1.455 | 41.4 | <0.001 |  |
| 3 Fish ~ Zpl + Pred + Depth | 1.254 | 49.6 | <0.001 |  |
| 4 Fish ~ Zpl + Pred + Depth + Dist | 1.247 | 49.9 | <0.001 |  |
| **5 Fish ~ Zpl + Pred + Depth + Dist + Hour** | **1.223** | **50.7** | **<0.001** | **4359** |
| II. Negative-binomial GAM |  |  |  |  |
| 1 Fish ~ Zpl | 0.15 | 28.4 |  |  |
| 2 Fish ~ Zpl + Pred | 0.03 | 39.6 | <0.001 |  |
| 3 Fish ~ Zpl + Pred + Depth | 0.149 | 47.2 | <0.001 |  |
| 4 Fish ~ Zpl + Pred + Depth + Dist | -0.156 | 47.7 | <0.001 |  |
| **5 Fish ~ Zpl + Pred + Depth + Dist + Hour** | **-0.165** | **48.3** | **<0.001** | **4359** |
| III. Gaussian GAM model on presence data only | |  |  |  |
| 1 Fish ~ Zpl | 0.253 | 37.7 |  |  |
| 2 Fish ~ Zpl + Pred | 0.221 | 45.8 | <0.001 |  |
| 3 Fish ~ Zpl + Pred + Hour | 0.217 | 46.8 | <0.001 |  |
| 4 Fish ~ Zpl + Pred + Hour +Depth | 0.208 | 49.2 | <0.001 |  |
| **5 Fish ~ Zpl + Pred + Hour +Depth + Dist** | **0.198** | **51.8** | **<0.001** | **2184** |
| IV. Binomial GAM on presence-absence data | |  |  |  |
| 1 Fish ~ Zpl | 0.103 | 20.6 |  |  |
| 2 Fish ~ Zpl + Pred | -0.032 | 30.4 | <0.001 |  |
| 3 Fish ~ Zpl + Pred + Hour | -0.04 | 31.1 | <0.001 |  |
| **4 Fish ~ Zpl + Pred + Hour +Depth** | **-0.208** | **43.3** | **<0.001** | **4359** |
| 5 Fish ~ Zpl + Pred + Hour +Depth + Dist | -0.208 | 43.3 | 0.456 |  |
