# Supplementary material for: Alien Invasions and the Game of Hide and Seek in Patagonia
Source: PLoS One. 2012 Oct 10;7(10):e44350. doi: 10.1371/journal.pone.0044350 (PMC3468591; doi:10.1371/journal.pone.0044350)
Supplement: Table S2. — (DOC) [file pone.0044350.s008.doc]

**Table S2. Test statistics of spatial autocorrelation of residuals** by means of Moran´s I (p-values) and Mantel test (r and p-values) for each final model: (I) quasi-poisson GAM; (II) negative-binomial GAM; (III) the Gaussian GAM model on presence data only; and (III) the binomial GAM on presence-absence data respectively.

| Model | Moran´s I (p) | Mantel (r) | Mantel (p) |
| --- | --- | --- | --- |
| I | 0.414 | 0.047 | 0.223 |
| II | 0.422 | 0.038 | 0.253 |
| III | 0.345 | 0.074 | 0.235 |
| IV | 0.407 | 0.02 | 0.364 |
