# Supplementary material for: Alien Invasions and the Game of Hide and Seek in Patagonia
Source: PLoS One. 2012 Oct 10;7(10):e44350. doi: 10.1371/journal.pone.0044350 (PMC3468591; doi:10.1371/journal.pone.0044350)
Supplement: Text S1. — (DOC) [file pone.0044350.s009.doc]

**Text SI**

*Threshold analysis of univariate time-series*

In order to investigate potential abrupt shifts in prey availability, predation risk and fish abundance over time (Figure 5E, F), we applied the Sequential Regime Shift Detection Method (STARS) [S1, S2] to normalized time-series (i.e., zero mean and unit variance) for each variable, respectively. The STARS algorithm is designed to detect statistically significant shifts in the mean level and the magnitude of fluctuations in time series by using modified two-sided Student’s t-tests. STARS can detect shifts at different time scales and magnitudes by varying the probability level of the tests and the cut-off length controlling the duration of regimes [S2]. In this study, we applied a significance-level of p=0.01 and cut-off lengths of 10±4 hours, in order to reduce the bias of arbitrarily setting a single cut-off length. Significant breakpoints were consistently found in all three series, regardless of cut-off lengths, corresponding to the decrease and increase in irradiance (PAR) at dusk and dawn, respectively (Figure 5E, F). More information on the STARS method and Excel add-in software is available online at [www.beringclimate.noaa.gov](http://www.beringclimate.noaa.gov/).

**Supporting References**

S1. Rodionov SN (2004) A sequential algorithm for testing climate regime shifts. Geophys Res Lett 31:L09204.

S2. Rodionov S, Overland JE (2005) Application of a sequential regime shift detection method to the Bering Sea ecosystem. Ices J Mar Sci 62:328-332.
